# The impact of the first and the second wave of the COVID-19 pandemic on vascular surgery practice in the leading regional center: a comparative, retrospective study

**DOI:** 10.1186/s40001-024-01720-y

**Published:** 2024-02-16

**Authors:** Katarzyna Stadnik-Zawalska, Julia Tomys-Składowska, Patryk Zawalski, Krzysztof Buczkowski, Arkadiusz Migdalski

**Affiliations:** 1 Department of Vascular Surgery and Angiology, Nicolaus Copernicus University in Toruń, Ludwik Rydygier Collegium Medicum in Bydgoszcz, 85-094 Bydgoszcz, Poland; 2 Jozef Brudzinski Provincial Children’s Hospital in Bydgoszcz, 85-667 Bydgoszcz, Poland; 3 Jan Biziel University Hospital No. 2 in Bydgoszcz, 85-168 Bydgoszcz, Poland; 4 Department of Family Medicine, Nicolaus Copernicus University in Torun, Ludwik Rydygier Collegium Medicum in Bydgoszcz, 85-094 Bydgoszcz, Poland

**Keywords:** COVID-19, Pandemic, Vascular surgery, Public health, Healthcare system, Health, Surgical treatment

## Abstract

**Background:**

We conducted an analysis of the vascular surgery regional center reorganization in response to the first and the second wave of the coronavirus disease-2019 (COVID-19) pandemic to see what lessons we learned from the first wave.

**Methods:**

The study included a total of 632 patients admitted to the vascular surgery department in three periods: March–May 2020, October–December 2020, and October–December 2019 as a control period.

**Results:**

In the pandemic periods the number of admitted patients decreased in relation to the control period. There was a reduction in performed procedures. We observed an increase in the ratio of less invasive procedures. There was a significant decline in hospitalization time in comparison to the control period.

**Conclusions:**

The reduction of scheduled admissions and procedures affected vascular centers all over the world. Minimally invasive procedures were more willingly performed to shorten the hospitalization time and reduce the patient's exposure to hospital infection. It allowed us to treat more patients during the second wave. Nevertheless, an increased number of vascular patients should be expected in the future, which will result from the failure to perform elective procedures during the pandemic.

## Background

The coronavirus disease 2019 (COVID-19) is caused by severe acute respiratory syndrome coronavirus 2 (SARS-CoV-2). The first reports of the disease come in December 2019 from Wuhan, Hubei Province, China [1].

The World Health Organization (WHO) declared COVID-19 as a Public Health Emergency of International Concern on January 30, 2020 [2]. On March 11, 2020, WHO declared COVID-19 as a pandemic, due to the rapid global spread and severe course of the disease [3].

In Poland, a state of pandemic was declared in March 2020. Simultaneously the first lockdown was introduced. All non-essential businesses and activities were ceased, and home confinement was required for everyone not involved in essential activities [4]. The COVID-19 pandemic has prompted a dramatic reorganization of the healthcare system. People's access to health care has significantly deteriorated. Dozens of public health facilities have been closed to patients not suffering from COVID-19. It has postponed or canceled many diagnostic and treatment elective hospitalizations, including planned surgeries in Poland [5]. Taking into consideration that patients with vascular diseases are especially prone to the development of complicated diseases if infected with SARS-CoV-2, it is particularly interesting to evaluate how the approach to that group of patients has changed during the COVID-19 pandemic [6].

Antoni Jurasz University Hospital number 1 in Bydgoszcz, Poland is the biggest hospital in the Kuyavian-Pomeranian Voivodeship inhabited by over 2.02 million people [7]. It is also a didactic center of the Nicolaus Copernicus University in Torun, Poland educating medical students, doctors, specialists, and employees of other medical professions. The Department of Vascular Surgery and Angiology is one of Poland's largest vascular surgery centers. It performs a full range of arterial and venous system procedures, including the most advanced and complicated surgeries. More than 1000 procedures are performed annually.

During the pandemic, our department was closed many times due to the COVID-19 infection among patients and personnel. Moreover, for several weeks it remained closed because rooms and beds were dedicated exclusively to patients with COVID-19 a "COVID-19 ward". This led to the cancellation of all vascular patients’ hospitalizations. Emergency cases were referred to lower-reference centers.

In our opinion, the impacts of COVID-19 on vascular surgery medicine, especially in Poland, have not been fully assessed yet. Although several publications have appeared on the impact of the COVID-19 pandemic on vascular surgery practice, they are from Western Europe or the United States. Results from neither Central Europe nor Poland were not published so far. In this context, we analyzed our department reorganization in response to the first and second waves of the COVID-19 pandemic. The analysis aimed to describe evolving strategies during the second wave of the pandemic, taking a lesson from the first wave.

Cardiovascular diseases are the leading cause of death [8]. It is necessary to assess the impact of the pandemic, lockdowns, and healthcare reorganization on the performance of vascular service provision and the changes needed to prepare for the post-pandemic time.

## Materials and methods

A retrospective registry of patients admitted to the Department of Vascular Surgery and Angiology Antoni Jurasz University Hospital number 1 in Bydgoszcz, Poland. Data were collected from 3 time periods: the period of the first wave of the pandemic (FWP) from March 1 to May 31, 2020, the period of the second wave of the pandemic (SWP) from October 1 to December 31, 2020, and the period before the pandemic (PBP) from October 1 to December 31, 2019 as a control period.

A total of 632 patients were included in the study (FWP: *n* = 188, SWP: *n* = 200, PBP: *n* = 244). We compared clinical and surgical data that were prospectively collected into a database, including pre-, intra-, and perioperative variables. The following were analyzed: length of hospitalization stay, the general condition of the patient on admission, the mode of admission (planned, urgent, acute), final diagnosis, and performed diagnostic and treatment procedures.

The general medical condition of patients on admission to the Department was determined by the American Hospital Association Guidelines for Releasing Information on the Condition of Patients. Patients were qualified as in good (vital signs stable and within normal limits, patient conscious), fair (vital signs stable and within normal limits, the patient is conscious, but may be uncomfortable), serious (vital signs un-stable and not within normal limits) and critical (vital signs unstable and not within normal limits, the patient may be unconscious) state [9]. Due to the small size of groups, serious and critical state patients were counted together.

Admissions were divided into planned (PA, scheduled admission), acute (AA, un-planned admission, surgery performed after detailed diagnostics and patient preparation, most often within a few days of admission), and urgent (UA, unplanned admission, emergency surgery, most often within 6 h of admission). A small group of patients was classified as "other admissions" (OA). They were mainly patients transferred from other wards after previously performed surgeries.

Planned admissions concerned cases of aortic aneurysms with a diameter over 55 mm in males and over 50 mm in females, high-grade carotid artery stenoses in asymptomatic patients, lower limb ischemia eligible for surgery except for critical ischemia, venous insufficiency and other arteries aneurysms or stenoses in asymptomatic patients. Acute admissions included large aortic aneurysms or aneurysms with a high risk of rupture, symptomatic carotid artery stenoses, and chronic limb-threatening ischemia (CLTI). Medical conditions that were treated with urgency included symptomatic and ruptured aneurysms, acute aortic syndromes, acute limb ischemia (ALI), and vascular traumatic injuries.

Final diagnoses ICD-10 codes were grouped into the following categories: ALI, carotid artery stenoses, aortic aneurysms (including abdominal, thoracic, and abdominothoracic), vascular graft infection, lower extremities artery disease (including CLTI), post-traumatic conditions, Leriche syndrome, venous insufficiency and thrombosis, other artery aneurysm and stenoses (i.e. renal, visceral, subclavian) and others—not classified elsewhere.

Performed procedures and operations were classified as follows: major and minor amputation, revascularization for lower extremity arterial disease (LEAD), carotid artery revascularization, endovascular aneurysm repair, open aorta repair, acute limb ischemia revascularization, angiography, venous revascularization, aorta revascularization, other aneurysms procedures, other artery revascularization (i.e. visceral, subclavian, renal) and others—not classified elsewhere.

The analysis of quantitative variables (i.e., expressed in numbers) was performed by calculating the mean, standard deviation, and median. The analysis of qualitative variables (i.e., not expressed in numbers) was performed by calculating the number and percentage of occurrences of each value. The comparison of qualitative variables in groups was performed using the chi-square test (with Yates' correction for 2 × 2 tables). The comparison of quantitative variables in the two groups was performed using the Mann–Whitney test. The analysis adopted a significant level of 0.05.

The registry data were collected using MS Office Excel^®^ and TIBCO Statistica 13.3.0 (Copyright © 2021 TIBCO Software Inc.) and analyzed using descriptive statistics.

The study design and methodology were developed based on similar, previously published studies describing the impact of the COVID-19 pandemic on various fields of medicine [10–13].

## Results

In the three analyzed periods, 632 patients were hospitalized. During the first wave of the pandemic (FWP), 188 patients were treated, which constituted 29.7% of all patients included in the study. During the second wave of the pandemic (SWP), the number of hospitalized patients increased slightly (*n* = 200, 31.6% of all patients included). In both analyzed periods, a decrease in the number of hospitalized patients was observed (FWP by 23%, SWP by 18%) (Table 1).

**Table 1:** Summarized description of patients included into the study

	FWP	*p* (vs. PBP)	SWP	*p* (vs. PBP)	PBP
Number of hospitalized patients, *n* (%) M male, F female	188	–	200	–	244
	M: 131 (69.6%)		M: 135 (67.5%)		M: 165 (69.6%)
	F: 57 (30.3%)		F: 65 (32.5%)		F: 79 (30.3%)
Percentage of all patients analyzed (%)	29.7	–	31.6	–	38.6
Urgent admission, *n* (%)	26 (13.8%)	ns	24 (12.0%)	ns	26 (10.7%)
Acute admission, *n* (%)	116 (61.7%)	< 0.00001	100 (50.0%)	0.000487	81 (33.2%)
Planned admission, *n* (%)	30 (16.0%)	< 0.00001	62 (31.0%)	0.000112	121 (49.6%)
Other admission, *n* (%)	16 (8.5%)	ns	14 (7.0%)	ns	16 (6.6%)
Age, mean (median) [years]	68.81 (69)	ns	68.69 (69)	ns	67.64 (69)
Hypertension, *n* (%)	142 (75.5%)	ns	136 (68.0%)	ns	175 (71.7%)
Diabetes mellitus, *n* (%)	52 (27.7%)	ns	68 (34.0%)	ns	72 (29.5%)
Smoking, *n* (%)	80 (42.6%)	ns	137 (68.5%)	< 0.00001	86 (35.2%)
Ischemic heart disease, *n* (%)	58 (30.9%)	ns	67 (33.5%)	ns	82 (33.6%)

FWP, first pandemic wave; SWP, second pandemic wave; PBP, period before pandemic; ns, non significance

The ratio of hospitalized women and men in all subgroups was similar. Women accounted for about one-third of those treated. The mean age of the patients in each group was similar and the median was identical in all subgroups (69 years).

**Fig. 1:**
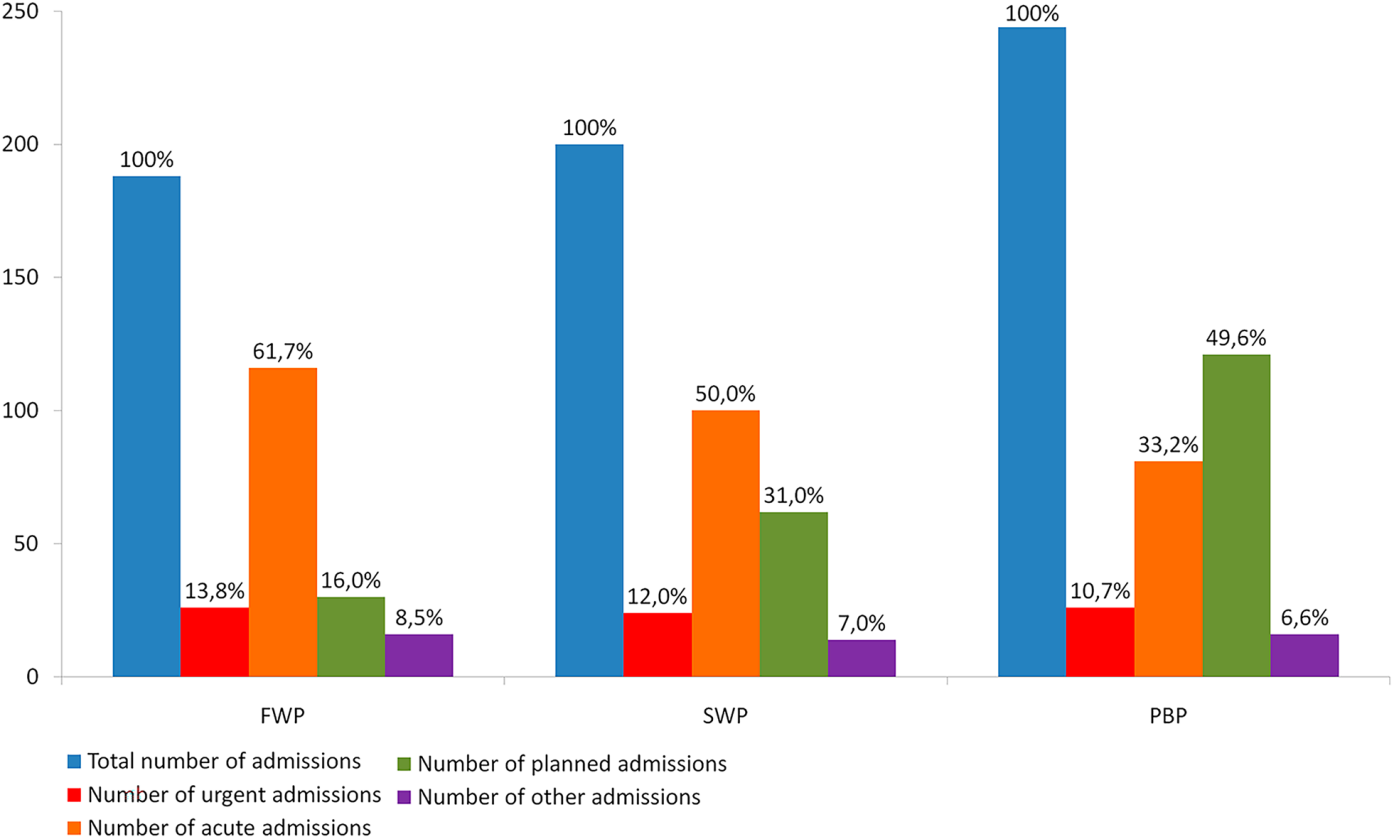
Types of admission to hospital, taking into account urgency (FWP, first wave of the pandemic; SWP, second wave of the pandemic; PBP, period before pandemic)

In the control period (PBP), almost half of the hospitalizations were scheduled admissions (PA). During the pandemic, the number of PA decreased significantly (fourfold reduction during FWP and twofold reduction during SWP) and acute admissions started to prevail. The number of urgent (UA) and other admissions (OA) in the analyzed periods were similar (Fig. 1).

**Fig. 2:**
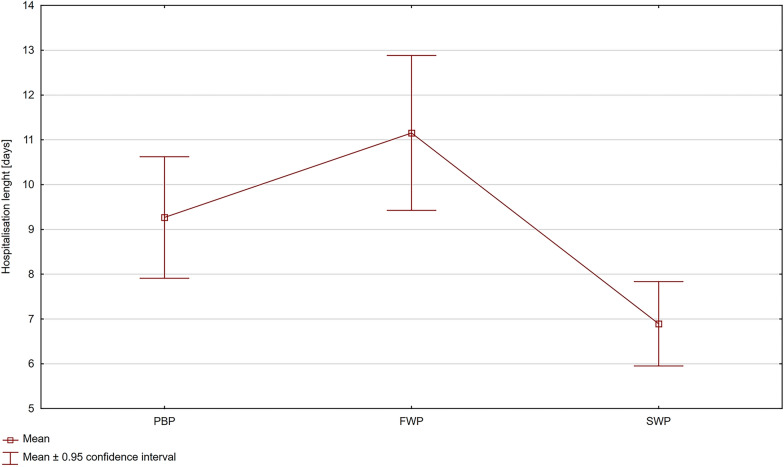
Mean hospitalization time graph (PBP, period before pandemic; FWP, first wave of the pandemic; SWP, second wave of the pandemic)

The mean length of hospitalization in the three analyzed periods was 9.1 days. During the first wave of the pandemic hospitalizations were noticeably longer than during the second wave (mean in FWP = 11.15 [days] vs. mean in SWP = 6.90 [days], *p* = 0.000001) and the control period (mean in FWP = 11.15 [days] vs. mean in PBP = 9.10 [days], *p* = 0.013504). During the second wave the trend was reversed, and hospitalization time was much shorter than in the control period (mean in SWP = 6.90 [days] vs. mean in PBP = 9.10 [days], *p* = 0.011087) (Fig. 2).

**Fig. 3:**
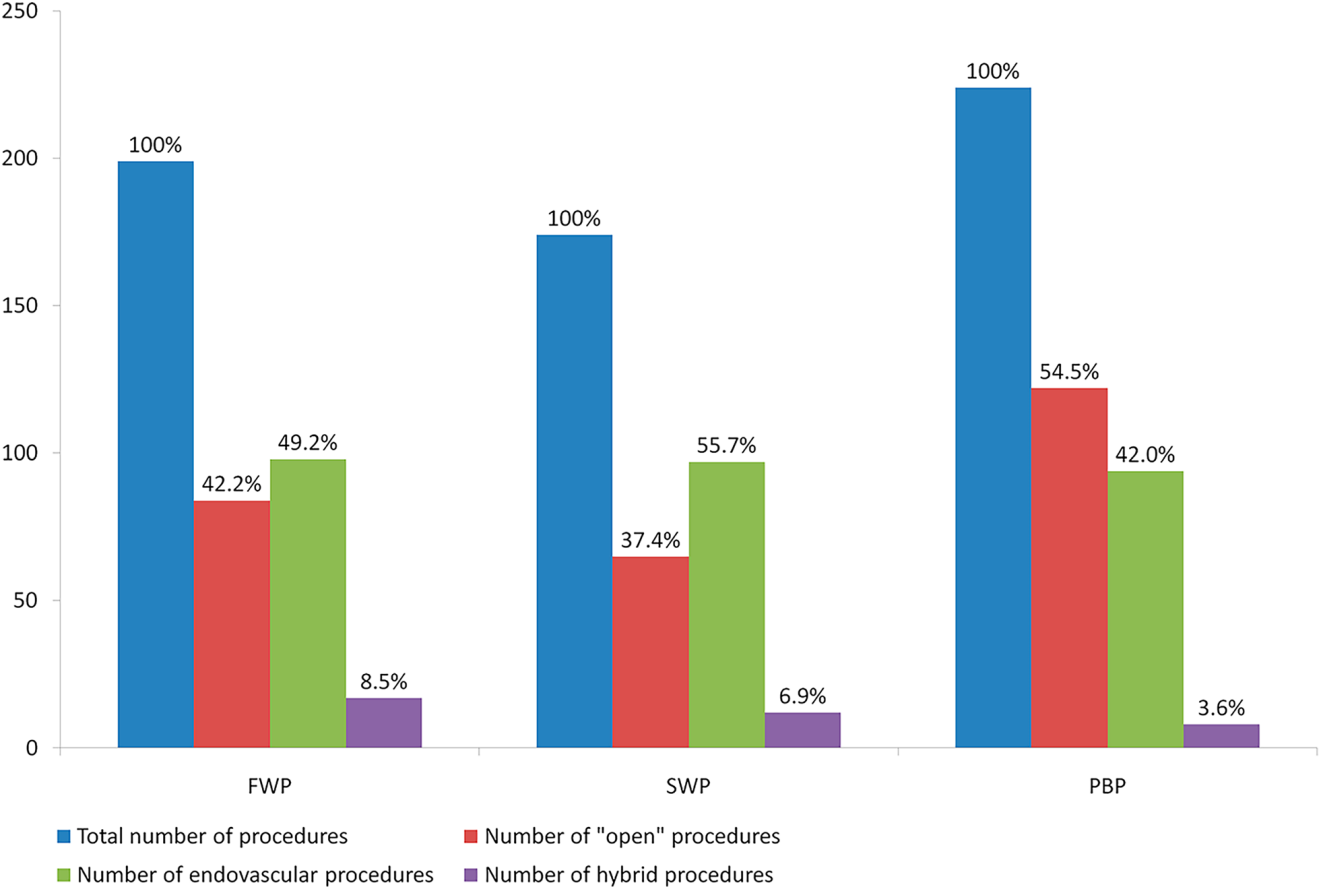
Performed procedures (PBP, period before pandemic; FWP, first wave of the pandemic; SWP, second wave of the pandemic)

In the three analyzed periods, 597 vascular procedures were performed. During FWP 199 procedures were performed in 156 patients. During SWP the number and mean of interventions performed per patient decreased. In both pandemic periods, a decrease in the number of procedures was observed compared to the control period (FWP by 11%, SWP by 22%) (Table 2) (Fig. 3).

**Table 2:** Number of vascular procedures performed

	FWP	*p* (vs. PBP)	SWP	*p* (vs. PBP)	PBP
Number of patients	188	–	200	–	244
Total number of procedures	199	–	174	–	224
Mean of procedures per patient	1.06	–	0.87	–	0.92
Number of “open procedures”, *n* (%)	84 (42.2%)	< 0.00001	65 (37.4%)	< 0.00001	122 (54.5%)
Number of endovascular procedures, *n* (%)	98 (49.2%)	0.0301	97 (55.7%)	0.008565	94 (42.0%)
Number of hybrid procedures, *n* (%)	17 (8.5%)	0.029161	12 (6.9%)	ns	8 (3.6%)
Number of patients without a vascular procedure, *n* (%)	32 (17.0%)	ns	48 (24.0%)	ns	41 (16.8%)

FWP, first pandemic wave; SWP, second pandemic wave; PBP, period before pandemic; ns, non significance

In the pandemic periods, an increase in the ratio of endovascular and hybrid procedures (FWP 57.7%, SWP 62.6% vs. PBP 45.6%) was detected. During the FWP, a significant decrease was found in the number of carotid artery revascularization (10 in FWP vs. 37 in PBP, a decrease of 72.97%, *p* = 0.000319), but an increase in the number of endovascular aneurysm repairs (EVAR) surgeries (38 in FWP vs. 23 in PBP, an increase of 39.47%, *p* = 0.01465) (Table 3).

**Table 3:** Diagnostic and treatment procedures

Procedure	FWP	*p* (vs. PBP)	SWP	*p* (vs. PBP)	PBP
Major amputation	17	ns	12	ns	11
Minor amputation	3	ns	4	ns	5
Revascularization for LEAD	59	ns	53	ns	61
Carotid artery revascularization	10	0.000319	17	ns	37
EVAR	38	0.01465	22	ns	23
OAR	12	ns	10	ns	15
Revascularization for ALI	27	ns	22	ns	26
Angiography	7	ns	6	ns	8
Other aneurysm procedures	4	ns	8	ns	8
Other artery revascularization (i.e., visceral, renal, etc.)	0	–	3	ns	4
Venous revascularization	2	ns	0	ns	4
Aorta revascularization	6	ns	8	ns	10
Others	14	ns	9	ns	12
Total	199	–	174	–	224

FWP, first pandemic wave; SWP, second pandemic wave; PBP, period before pandemic; LEAD, lower extremity arterial disease; EVAR, endovascular aneurysm repair; OAR, open aortic aneurysm repair; ALI, acute limb ischemia; ns, non significance

The medical state of patients on admission to the department was similar in both pandemic periods and during the control time. Most of the patients admitted to the department on a planned or acute mode were in a good or fair state. In serious and critical condition were mainly patients suffering from ruptured aneurysm and after trauma admitted in urgent mode (Table 4).

**Table 4:** Medical state of patients on admission

Medical state	FWP	*p* (vs. PBP)	SWP	*p* (vs. PBP)	PBP
Good, *n* (%)	160 (85.1%)	ns	174 (87%)	ns	216 (88.5%)
Fair, *n* (%)	21 (11.2%)	ns	20 (10%)	ns	24 (9.8%)
Serious and critical, *n* (%)	7 (3.7%)	ns	6 (3%)	ns	4 (1.6%)

FWP, first pandemic wave; SWP, second pandemic wave; PBP, period before pandemic; ns, non significance

The cross-section of final diagnoses did not change throughout the pandemic waves (Table 5). During the FWP, we observed a significant decrease in patients with carotid artery stenoses (72.22%), and an increase in acute limb ischemia (ALI) 42.86%) and aortic aneurysm disease (25%).

**Table 5:** Final diagnosis

Diagnosis	FWP	*p* (vs. PBP)	SWP	*p* (vs. PBP)	PBP
Carotid artery stenoses	10	0.002747	21	ns	36
ALI	30	0.028011	29	ns	21
Aortic aneurysms	60	0.005088	37	ns	48
Other artery aneurysms	7	ns	13	ns	8
Other artery stenoses (i.e., renal, visceral, subclavian)	0	–	1	0.021608	11
Vascular graft infection	4	ns	3	ns	2
LEAD	55	ns	72	ns	91
Complications after previous vascular procedures	6	ns	9	ns	12
Post-traumatic conditions requiring vascular interventions	5	ns	2	ns	3
Leriche syndrome	5	ns	6	ns	5
Venous thrombosis	1	ns	1	ns	2
Others	5	ns	6	ns	5

FWP, first pandemic wave; SWP, second pandemic wave; PBP, period before pandemic; ns, non significance; ALI, acute limb ischemia; LEAD, lower extremity arterial disease

The decreased number of patients diagnosed with carotid artery stenoses concerned asymptomatic patients. The number of symptomatic ones was 6, 8, and 6 during FWP, SWP, and PBP, respectively. The symptomatic to asymptomatic ratio was, respectively, 1.50 (FWP), 0.62 (SWP), and 0.20 (PBP).

During the pandemic, the absolute number of patients admitted because of LEAD decreased regarding to the control period (65.5% during FWP and 26.4% during SWP) but without statistical significance. Patients with LEAD constituted 29.3% in FWP, 36.0% in SWP, and 37.3% in PBP of all admitted patients, respectively. The majority of patients treated during the pandemic suffered from limb ischemia in the III and IV stage of the Fontaine Classification [14].

During FWP, the number of amputations was higher (total amputations: 20 in FWP vs. 16 in PBP and major amputations 17 in FWP vs. 11 in PBP) but without statistical significance. The number of patients diagnosed with acute limb ischemia (ALI) was 30, 28, and 21 during FWP, SWP, and PBP, respectively. The amputation-to-revascularization ratio due to ALI treatment was 4:21, 4:18, and 0:19 during FWP, SWP, and PBP, respectively. The other patients with ALI (4 in FWP and 3 in SWP) were treated conservatively or disqualified from treatment due to their severe general condition. Among patients with ALI in SWP four were diagnosed with COVID-19, one was convalescent, and one patient’s test result was inconclusive. In a group of patients with confirmed COVID-19, a higher rate of revascularization failure and the necessity for amputation were observed. Patients admitted during FWP were not tested for COVID-19 or they presented negative results.

## Discussion

The COVID-19 pandemic has affected our vascular surgery and angiology department in multiple ways. The pandemic had an impact on the number of hospitalizations and resulted in a decrease in scheduled admissions. This was in line with the global trend not only in vascular but in other branches of surgery as well [13, 15]. During SWP, the decrease in scheduled admissions was lower than during FWP (48.76% vs. 75.21%). This may be due to the staff becoming acquainted with the procedures applicable during the pandemic. In the United States, during the early pandemic (April 2020) 98% of vascular surgery practices limited elective admissions, while during the late pandemic (August 2020) only 19% [16]. Researchers from Northern Italy noticed an 18% increase in the number of procedures performed during phase 2 of the pandemic (May–June 2020) compared with phase 1 (March–April 2020) [12]. General surgeons from this country found a 25% decrease in the number of surgeries during the pandemic compared to the previous period. Similarly to our observation, patients operated for emergency reasons was significantly older than in the period before the pandemic [13]. The reduction of scheduled admissions led to a decreased number of performed procedures and surgeries. In 2020 Ng et al. conducted a survey among vascular surgeons from all over the world about the procedures performed during the pandemic. Results showed that 90.9% of the units had postponed scheduled admissions. The majority of cases canceled were varicose vein surgery, revascularization for claudication, “small” or asymptomatic aortic aneurysms, dialysis access, and asymptomatic carotid stenoses procedures [17].

In our department, we also observed a statistically significant decrease in the number of patients admitted with asymptomatic carotid artery stenoses. Carotid stenoses are considered to be responsible for 10–15% of ischemic strokes [18]. Current guidelines recommend performing carotid revascularization within 14 days after symptoms onset to decrease the risk of recurrent cerebrovascular accidents [19]. Postponing procedures in these patients would not be reasonable. Hospitalizations of patients with asymptomatic carotid stenoses were also postponed in other countries. In France during the first lockdown of the COVID-19 pandemic, a 58% decrease in carotid revascularization procedures was observed, mostly concerned asymptomatic patients [20]. Asymptomatic carotid stenosis is a risk factor for stroke, myocardial infarction, and mortality. The latest cohort studies comparing carotid revascularization versus medical therapy in patients with asymptomatic carotid stenosis showed that 30-day stroke risk reaches 0.6% without surgical intervention. Long-term risk of ipsilateral stroke totals from 1.98 to 5.5% on pharmacological therapy and decreases to 0.65% after carotid endarterectomy [21].

The consequences of postponing lower limb revascularization procedures are difficult to determine. There is no evidence for the effectiveness of non-revascularization methods in the treatment of CLTI. Progressing walking impairment in earlier stages can be treated with verapamil, statins, antiplatelet agents, prostanoids, and non-pharmacologically by exercise therapy [22]. The Claudication: Exercise Versus Endoluminal Revascularization (CLEVER) study demonstrates that for patients with claudication, supervised exercise therapy provides a superior improvement in treadmill walking performance compared to both primary stenting and optimal medical care (home walking and cilostazol) over 6 months. This benefit was associated with an improvement in self-reported walking distance, an increase in HDL fraction of cholesterol, and a decrease in fibrinogen [23]. Other vascular surgery units, likewise our department, had reported admitting for surgery fewer patients with LEAD but with a more severe clinical presentation during the pandemic. It resulted in palliative care and primary amputations in some cases [24]. This is confirmed by the latest data from Brazil, where a significant increase in lower limb amputations has been recorded during the pandemic [25]. Direct research on LEAD progression is essential, both in asymptomatic and symptomatic patients whose procedures were canceled or postponed due to the COVID-19 pandemic.

Unexpectedly, in our results, the number of patients with aortic aneurysm procedures was significantly higher during FWP. The number of open aorta repair (OAR) procedures was similar to other periods, but the number of endovascular aneurysm repair (EVAR) procedures increased considerably. EVAR reduces the average hospital stay by 296.75 h compared to OAR [26]. The increased number of minimally invasive aneurysm operations resulted from a desire for faster discharge. Other data were obtained from centers in other countries. German surgeons observed a significant (− 25.5%) decrease in the number of patients with abdominal aortic aneurysms during the first wave of the pandemic. Similarly to our center, the number of cases treated endovascularly increased during the all waves of pandemic. Interestingly, a significant increase (22.2%) in the number of ruptured aneurysms has been observed during the pandemic [27].

The pandemic has shifted the treatment profile towards less invasive procedures. During the control phase “open procedures” accounted for 54% of all procedures. In both the first and the second wave of the pandemic endovascular and hybrid procedures were performed more often than open procedures. The importance of endovascular methods in vascular surgery has increased in recent years. In Guez et al. research endovascular treatment of peripheral artery disease (PAD) in the years 2011–2016 increased by 25% [28]. In our department, the year-to-year increase in endovascular treatment was 13.7% (PBP 42.0% vs. SWP 55.7%). The percentage rise resulted from a trend of change towards endovascular therapy, however, the pandemic period significantly accelerated this process. Referring to the meta-analysis by Ting et al. comparing outcomes of open surgery and endovascular methods of PAD treatment, the latter presents more advantages. In comparison to open surgery, endovascular treatment is characterized by less complication, lower mortality rate, lower amputation risk, and shorter hospital stay [29]. The role of endovascular therapy in the pandemic period is even more important. A shorter hospital stay reduces the risk of nosocomial infection for both the patient and the medical staff, in case of operating COVID-19-positive patients. In addition, the ability to perform procedures under local anesthesia decreases the chance of postoperative complications, thereby increasing ICU resources such as bed availability [30, 31]. The pandemic period contributed to accelerating progress in the use of minimally invasive procedures. During the acute phase of the COVID-19 pandemic in Italy, endovascular procedures were chosen with increased frequency. Among the advantages of these methods, the vascular surgery unit included the ability to perform surgery under local anesthesia [32]. The pandemic has forced surgeons to change algorithms and adapt treatment to current resources. According to the COVER study, 60.4% of units selected endovascular methods as the first choice of limb ischemia treatment during the pandemic [33]. Examples from other centers prove the shift towards the endovascular approach and its use in circumstances where it was previously not the first-line treatment. The constant development of technology allows open surgery to be replaced by less invasive procedures. Robot-assisted procedures are also starting to appear in vascular surgery. These techniques have found applicability in the treatment of PAD, fenestrated endovascular aortic repair, carotid artery stenting, and transfemoral renal and mesenteric intervention. They are not yet common due to technical difficulties [34]. However, the ability to replace some medical staff members with technology may also be advantageous during a critical period such as a pandemic.

During the first phase of the pandemic, there was a significant increase in diagnosed acute limb ischemia (ALI). The association between ALI and SARS-CoV-2 is well documented and caused by a hypercoagulable condition and hyperimmune systemic response provoked by pneumonia [35]. The COVID-19 disease is also known as a risk factor for arterial and venous thrombosis [36]. As reported, SARS-CoV-2 infection leads to increased values of D-dimers, fibrinogen, and von Willebrand factor (vWF). A short prothrombin time (PT) and activated partial thromboplastin time (aPTT) is only observed in some patients. In severe cases, a condition resembling disseminated intravascular coagulation (DIC) develops [37, 38]. As described in Wool and Miller's article, in 5.0–41.7% of patients, thrombocytopenia is found. The authors observed elevated immature platelet fraction (IPF) in COVID-19 cases, and also in patients without thrombocytopenia. This may explain the mechanism of hypercoagulability due to the higher activity of immature platelets and their easier aggregation [37]. Research by Cohen et al. confirmed that IPF is positively correlated with the severity of the disease and may be used as its indicator [39]. Pneumonia caused by COVID-19 is associated with vascular endothelium damage and its dysfunction. Apoptosis of infected endothelial cells triggers pro-coagulant processes [38]. The unbalanced immune response in severe patients may lead to the overactivation of neutrophils. This process triggers pro-thrombotic pathways. As suspected, the cytokine storm, uncontrolled complement activity, and NETosis (program for the formation of neutrophil extracellular traps) may be responsible for blood clotting. This mechanism is also known as immune thrombosis [40]. Ali and Spinler’s article lists deep vein thrombosis and pulmonary embolism as the most common vascular coagulopathies associated with COVID-19 [40]. Thrombotic events such as ALI, ischemic stroke, and myocardial infarction are observed as well [40–42].

A reduction in the length of hospitalization during the SWP compared to the PBP is noticeable. The reason for the shorter hospital stay was an attempt to reduce the risk of nosocomial transmission of COVID-19. The [42], research shows that hospital-onset COVID-19 infections (HOCIs) reach about 12–15% of all COVID-19 cases in healthcare institutions. At the culmination of the pandemic, the risk rises to 16.2% [42]. The noticeable extension of hospitalization time during FWP is probably because mostly acute and urgent mode patients were admitted, in a worse general condition, and required longer convalescence after the procedure. A similar observation was made in Italy, where the length of hospital stay increased from 3.3 ± 2.7 days in pre-pandemic to 5.3 ± 3.9 in the pandemic era (*p* = 0.004) [43].

The pandemic allowed us to gain knowledge on how to act in a crisis. After analyzing the data from the first wave, we concluded the patient's eligibility for surgery. Similar changes were also forced by the pandemic in other areas of surgery [13]. Endovascular surgeries were performed more frequently. As a result, in subsequent phases of the pandemic, hospitalization time was reduced and the risk of nosocomial SARS-CoV-2 infection decreased. Also, we were able to perform more short-time admissions, thereby the waiting time for treatment was reduced. The procedures deferred earlier due to the first wave were performed as well. This improvement was limited by the short period between the waves and by a concentration of human and financial resources on dealing with the current pandemic threat. Also, such a rapid development of the pandemic in Poland was unexpected. From March 1 to May 31, 2020, there were 23,571 new cases and 1061 deaths. The second wave was more widespread, as 1,206,507 new cases and 26,128 deaths were noted from October 1 to December 31, 2020 [44].

## Conclusions

The descriptive nature of the study was aimed to emphasize the experience of a single vascular surgery center in response to the COVID-19 pandemic. The pandemic had a significant impact on healthcare systems and daily surgical practice worldwide. Its consequences are still being experienced to this day.

Vascular surgery and angiology units were affected in multiple ways. The described department’s situation is not unique, as multiple vascular surgery units around the world experienced the same changes. There was an overall reduction in vascular activity including inpatient procedures and elective admissions.

Despite the limitations, we managed to learn a valuable lesson from the first wave of the pandemic, and we did better in the next waves. Nevertheless, an increased number of vascular patients should be expected in the future, which will result from the failure to perform the necessary elective procedures during the pandemic as well as from the studied effect of the coronavirus infection on thromboembolic complications.

Lockdowns and limited access to healthcare caused by the pandemic have generated a "health debt". Its repercussions may be felt even years after the pandemic ends. More research is indispensable to evaluate the impact on patients with vascular diseases. It is required to develop procedures and workflows to confine the long-term effects of the COVID-19 pandemic on these patients’ state of health.

## Data Availability

The dataset used for this study is in the custody of the corresponding author and will be made available upon a reasonable request.

## Abbreviations

COVID-19: Coronavirus disease-2019
SARS-CoV-2: Severe acute respiratory syndrome coronavirus 2
WHO: World Health Organization
FWP: First wave of the pandemic
SWP: Second wave of the pandemic
PBP: Period before the pandemic
PA: Planned admissions
AA: Acute admissions
UA: Unplanned admissions
OA: Other admissions
CLTI: Chronic limb-threatening ischemia
ALI: Acute limb ischemia
LEAD: Lower extremity arterial disease
OAR: Open aorta repair
EVAR: Endovascular aneurysm repair
vWF: Von Willebrand factor
PT: Prothrombin time
aPTT: Activated partial thromboplastin time
NETosis: Program for the formation of neutrophil extracellular traps
